# The complete mitochondrial genomes of *hycleus cichorii* and *hycleus phaleratus* (Coleoptera: Meloidae)

**DOI:** 10.1080/23802359.2018.1431066

**Published:** 2018-02-07

**Authors:** Yuanming Wu, Yangyang Liu, Xiangsheng Chen

**Affiliations:** a Institute of Entomology, Special Key Laboratory for Development and Utilization of Insect Resources, Guizhou University, Guiyang, Guizhou, P.R. China;; b Department of Parasitology, Laboratory of Pathogenic Biology, Basic Medical College, Guizhou Medical University, Guiyang, Guizhou, P.R. China

**Keywords:** *Hycleus cichorii*, *hycleus phaleratus*, mitochondrial genomes, complete sequence, molecular phylogeny

## Abstract

The *Hycleus cichorii* and *Hycleus phaleratus* are two species of medicinal meloids widely distributed in southwest of China. We sequenced an annotated the complete mitochondrial genomes of *H. cichorii* and *H. phaleratus*, and the mitogenomes are 15,847 and 16,004 bp in length, respectively. Every mitochondrial genome encodes 13 proteins, 2 ribosomal RNAs, 22 tRNAs, and a control region with the identical arrangement to other beetles. The preliminary phylogenetic analysis with mitochondrial genomes of nine meloid species further confirmed the status of these two species.

Blister beetles have a long history in human medicine for its irritant property (Bologna 1991; Tan et al. 1995). Their released oils contain the skin irritant cantharidin, which is a defensive terpenoid and sexual pheromone. The mitochondrial genomes of meloids were described and utilized to infer their phylogenetic implications (Du et al. 2016; Yuan et al. 2016; Du et al. 2017). However, some relationships among this family were unresolved due to the limitation of available mitochondrial information. In this study, we present the complete mitochondrial genomes of *H. cichorii* and *H. phaleratus*, which would be a significant increase in further study of mitochondrial genome architecture and meloid phylogenetics. Specimens were collected in Luodian, Guizhou, China, and disposed in Institute of Entomology/Special Key Laboratory for Development and Utilization of Insect Resources, Guizhou University. The high-throughput sequencing method was used to obtain the complete mitochondrial genomes.

The complete mitochondrial genome of *H. cichorii* (accession no. MF491388) and *H. phaleratus* (accession no. MF491389) are 15,847 and 16,004 bp in length, respectively. Every mitochondrial genome encodes the typical 37 genes including 13 protein-coding genes, 22 transfer RNAs, and 2 ribosomal RNAs. The A + T contents of two mitochondrial genomes are 73.0% and 69.9%, respectively. All 13 protein-coding genes used typical ATN start codons, including eight Met (ATA & ATG) and five Ile (ATT). Conventional stop codons could be assigned to most of the PCGs, while *cox1*, *cox2*, *nad5,* and *nad4* terminated with incomplete stop codon T. All tRNAs could be folded into the typical clover-leaf structure, except *trnS(AGN)* lacked a dihydrouridine (DHU) arm, which was replaced by a simple loop. The lengths of *rrnL* were determined to be 1,281 and 1,276 bp in *H. phaleratus* and *H. cichorii* respectively, and the lengths of *rrnS* were 793 bp in both species. The 1,129 and 1,128 bp control regions of *H. phaleratus* and *H. cichorii* mitogenome were located between *rrnS* and *trnI*.

Phylogenetic analysis was performed based on 13 protein-coding genes of nine meloid species, by using Bayesian Inference. The result further confirmed that *H. phaleratus* and *H. cichorii* were closely related to *H. marcipoli* and *H. chodschenticus*, and all *Hycleus* species were recovered as a monophyly to sister to *M. aulica* (Figure 1). Besides, the relationships among Meloidae were congruent with the previous study (Du et al. 2017).

**Figure 1.**
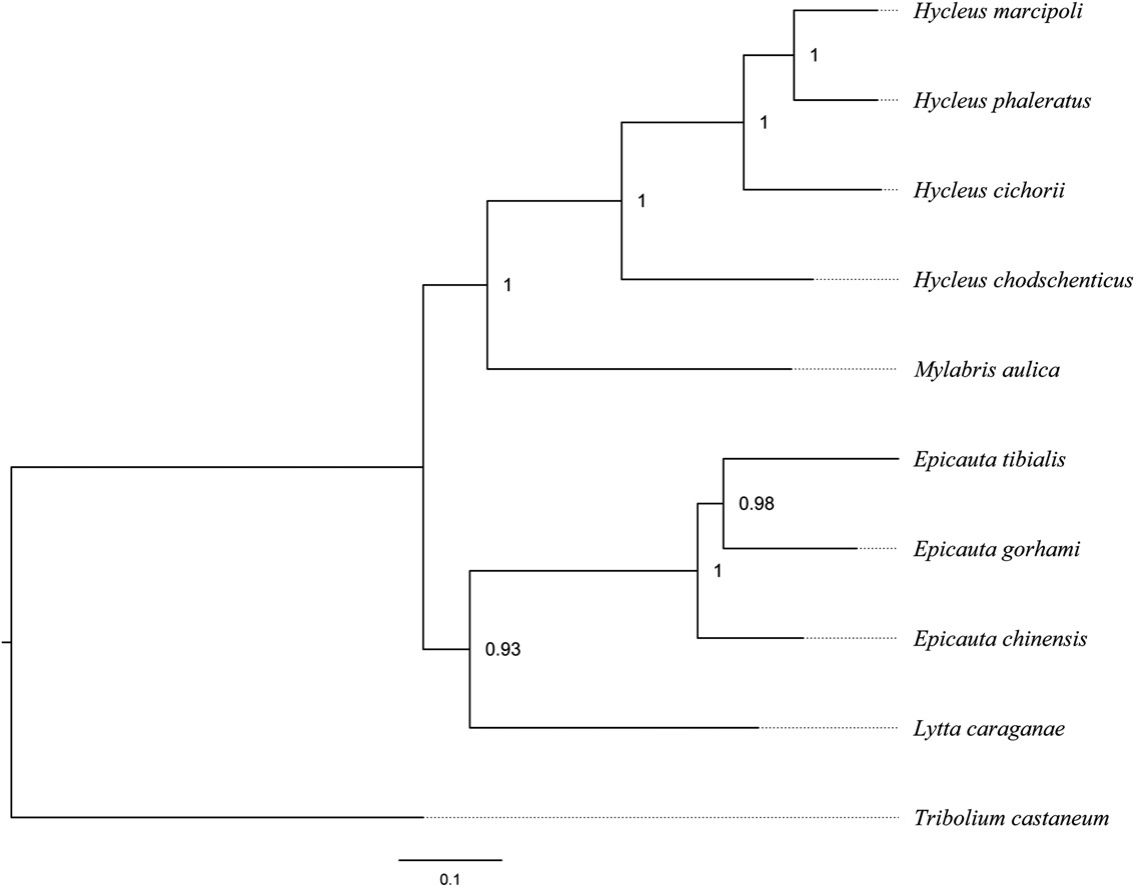
The Bayesian phylogenetic tree of nine meloids was constructed using the dataset of 13 protein-coding genes, with the tenebrionoid *Tribolium castaneum* employed as the outgroup. The numbers abutting nodes refer to Bayesian posterior probabilities. Sequence data used in this study are the following: *Hycleus marcipoli* (KX161857), *Hycleus chodschenticus* (KT808466), *Mylabris aulica* (KX161860), *Epicauta chinensis* (KP692789), *Epicauta tibialis* (KX161855), *Epicauta gorhami* (KX161854), *Lytta caraganae* (KX161859), *Tribolium confusum* (NC_026702).
